# Business model configuration and dynamics for technology commercialization in mature markets

**DOI:** 10.1108/BFJ-03-2017-0125

**Published:** 2017-11-06

**Authors:** Serena Flammini, Gabriella Arcese, Maria Claudia Lucchetti, Letizia Mortara

**Affiliations:** 1 Centre for Technology Management, Institute for Manufacturing, University of Cambridge, Cambridge, UK; 2 Department of Business Studies, Roma Tre University, Rome, Italy; 3 Ionian Department of Law, Economics and Environment, University of Bari Aldo Moro, Bari, Italy

**Keywords:** 3D printing, Open business model, Business model innovation, Technology commercialization, Emerging technologies, Established industries

## Abstract

**Purpose:**

The food industry is a well-established and complex industry. New entrants attempting to penetrate it via the commercialization of a new technological innovation could face high uncertainty and constraints. The capability to innovate through collaboration and to identify suitable strategies and innovative business models (BMs) can be particularly important for bringing a technological innovation to this market. However, although the potential for these capabilities has been advocated, we still lack a complete understanding of how new ventures could support the technology commercialization process via the development of BMs. The paper aims to discuss these issues.

**Design/methodology/approach:**

To address this gap, this paper builds a conceptual framework that knits together the different bodies of extant literature (i.e. entrepreneurship, strategy and innovation) to analyze the BM innovation processes associated with the exploitation of emerging technologies; determines the suitability of the framework using data from the exploratory case study of IT IS 3D – a firm which has started to exploit 3D printing in the food industry; and improves the initial conceptual framework with the findings that emerged in the case study.

**Findings:**

From this analysis it emerged that: companies could use more than one BM at a time; hence, BM innovation processes could co-exist and be run in parallel; the facing of high uncertainty might lead firms to choose a closed and/or a familiar BM, while explorative strategies could be pursued with open BMs; significant changes in strategies during the technology commercialization process are not necessarily reflected in a radical change in the BM; and firms could deliberately adopt interim strategies and BMs as means to identify the more suitable ones to reach the market.

**Originality/value:**

This case study illustrates how firms could innovate the processes of their BM development to face the uncertainties linked with the entry into a mature and highly conservative industry (food).

## Introduction

Well-established industries represent core sectors of the international economy (Caiazza, 2015). The configuration of these industries presents challenges for new entrants. The food and drink industry, for instance, is known to be historically characterized by well-established dominant designs, great fragmentation and low-tech innovation rates (Manzini *et al.*, 2016). It comprises few very powerful multinational incumbents, but most companies in the sector are small and medium enterprises (SMEs) (about 99 per cent of the market according to Food Drink Europe, 2015). Hence, the food industry is regarded as a complex industry where potential new entrants have to face high entry barriers and need to cope with the constantly changing demands of the customers (Bigliardi and Galati, 2013a, b; Caiazza, 2015; Chesbrough and Crowther, 2006; Fortuin and Omta, 2009; Manzini *et al.*, 2016; Swaminthan, 1998). To face these challenges, new entrants need to innovate. Technological innovations that can bring great changes into the industry and would help firms meet and exceed the customers’ expectations, could provide a great opportunity for new entrants to access the sector. 3D printing technologies could indeed provide such advantages and support an increased rate of product customization (Jia *et al.*, 2016). However, introducing these technologies and innovations into a mature and potentially conservative market brings high uncertainty for new entrants. Scholars have highlighted that the capability to innovate the commercialization processes, through collaboration with others and through the identification of the most suitable approaches (i.e. strategy and business models (BMs)) to commercialize the new products, might increase the chances to penetrate a sector. For new entrants, technology development and commercialization are often impossible to achieve in isolation (Maine and Garnsey, 2006). Collaborations with partners are not just needed for developing an innovation (Chesbrough, 2003a, b), but also to assemble the complementary elements (e.g. other products, technologies or services) necessary for delivering value from the technology to customers (Vanhaverbeke and Chesbrough, 2014). Hence, the capability to innovate the BM is a key skill to enable new entrants to commercialize their technologies (Massa and Tucci, 2013) through which a firm can maintain viability and ensure they have a unique competitive advantage in a mature industry (Cortimiglia *et al.*, 2016). Therefore, both the firm’s approach to openness and to the innovativeness of the BM can provide a great contribution to the firm’s ability to actualize its strategy and, therefore, commercialize its innovation.

However, despite the importance of these innovation processes in enabling the commercialization of an emerging technology in an established market, we still lack a complete understanding of the BM dynamics which support this commercialization process. So, following Caiazza (2015) and Probert *et al.* (2013), this paper asks “how do companies change the BM when they attempt to exploit an emerging technology in an established industry?”.

In response to this question, we started assembling a framework that describes what we know about the BM development in the commercialization process of emerging technologies, linking theoretical concepts from the technology commercialization literature with studies on strategy, (collaborative) BMs and BM innovation. Then we observed an empirical case study based on the experience of IT IS 3D (a company that has started to exploit 3D printing in education and is moving towards the food preparation industry), which was used to improve the framework. From this case study we observed that: companies could use more than one BM at a time; hence, the BM innovation processes (design and reconfiguration) could co-exist and be run in parallel; the facing of high uncertainty might lead firms to choose a closed and/or a familiar BM, while explorative strategies could be pursued with open BMs; significant changes in strategies during the technology commercialization process (e.g. opening new markets) are not necessarily reflected in a radical change in the BM; and firms could deliberately adopt interim strategies and BMs as a means to identify the more suitable ones to reach the market.

By highlighting how new entrants innovate their business model innovation (BMI) process to enter into an established environment, this work helps both practitioners and academics to understand how new ventures cope with the uncertainty linked to the penetration of an established industry.

## Background

### Innovation for technology commercialization in established industries: evidence from the food industry

The food industry is a mature industry of key importance for the global economy. It generates more than four trillion US dollars in annual retail sales (Plunkett Research Ltd., 2017) and in Europe it is the largest manufacturing sector, not only in terms of turnover (i.e. €1,244 billion), but also in terms of employment (i.e. 4.2 million people) and value added (i.e. 1.8 per cent of the European value added) (Food Drink Europe, 2015). At the same time, the food industry is also characterized by a high level of heterogeneity in the types of enterprises, the types of production, and in their retail and distribution organizations (Pellegrini *et al.*, 2014). The composition of this industry is characterized by the presence of just a few large incumbents while the majority of the firms in the sector are SMEs, which in Europe represent 99.1 per cent of the sector.

Given its complexity, new entrants have to face high barriers to entry and hence, need to acquire the capability to respond promptly to the constantly changing needs of the customer (Bigliardi and Galati, 2013a, b; Manzini *et al.*, 2016) by becoming more innovative.

Typically, new entrants in the food industry have been mostly focusing on product innovations, trying to meet and exceed the customers’ expectations (Bigliardi and Galati, 2013a, b; Manzini *et al.*, 2016). 3D printing technologies are emerging digital technologies that can provide an answer to this need. Personalization and customization of products is a main trend among customers' demands for consumer goods, and 3D printing technologies have the potential to allow the mass-personalization of food and packaging (Jia *et al.*, 2016; Sun *et al.*, 2015). Hence, they have the potential to disrupt the existing food value chain, representing potential business opportunities but also great uncertainty for new entrants (Porter, 1980). The technology commercialization literature suggests that ventures willing to commercialize new process technologies need to be able to innovate and also adapt the technology commercialization processes to respond to the obstacles and uncertainties along the way (Maine *et al.*, 2012). Collaboration with other actors and the identification of the most suitable strategies and BMs to commercialize their innovations can be of strategic importance (Vanhaverbeke and Chesbrough, 2014; Casadesus-Masanell and Ricart, 2010). However, despite the identification of the importance of these process innovations, there is still the need to clarify the dynamics that lead companies to access a mature industry based on the commercialization of an emerging technological innovation (Probert *et al.*, 2013; Caiazza, 2015).

### The commercialization of emerging technologies: the link between commercialization strategy and BMs

Past literature has taken a dual view of technology commercialization. Some see this as part of the innovation process (Burgelman, *et al.*, 2004), others as part of the diffusion of innovations into the market (e.g. Nambisan and Sawhney, 2007; Nerkar and Shane, 2003). Datta *et al.* (2015) identified six main steps that lead the technological innovations into the market, based on three main phases of the innovation process: ideation, development and deployment (Teece, 1986; Teece *et al.*, 1997). Whilst “Ideation” concerns idea generation; in the “Development” phase the commercialization strategies are defined. Finally, the “Deployment” phase sees the firm’s strategy actualization through the definition of a BM (i.e. a BM explains the target market and the appropriate value proposition, the resources needed and the placement of a firm in a value chain – (Casadesus-Masanell and Ricart, 2010).

The strategy literature suggests contrasting commercialization strategies with new technologies. As highlighted by Osterwalder and Pigneur (2010), there are several types of market choices from which an entrepreneur can choose, such as mass, niche, segmented, diversified and multi-sided. As initially highlighted in the studies on disruptive innovation, new ventures – which aim to commercialize innovation based on change to “emerging technologies” with the potential to be exploited within the next ten years (Keenan, 2003) – can easily achieve their competitive advantage without the pressure of the incumbent firms by taking up opportunities where the markets are not yet completely formed (Christensen, 1997; Davidow, 1986; Christensen and Rosenbloom, 1995) – i.e. they should prefer niche strategies. Nerkar and Shane (2003) take the opposite perspective, and recommend that new ventures develop intellectual properties in a way that could suit a wide range of markets. Later, Lubik and Garnsey (2016) suggested that radical, generic technology-based ventures should select mainstream markets to target rather than niche markets, due to the presence of incumbent firms that can provide the resources needed by the new venture. The choice of the most suitable exploitation strategy according to Grant (2016) is instead linked to the resources and capabilities of the innovator firm. Specifically referring to established industries, he suggests that firms should exploit innovations through differentiation strategies (Grant, 2016).

For new ventures, difficulties lie in being able to choose among the great number of market options available (Maine *et al.*, 2012), and in the considerable challenges that they face to gain access to the resources that are useful for their creations. Whatever the strategic choice, among the possible uncertainties in the commercialization of a technology, there is the commercialization of other complementary technologies in the present or in the future (Teece, 2010). Hence, technology-based new ventures, which are typically characterized by scarce resources and capabilities, are keen to reduce the risks generated by uncertainties through strategies that rely on the support of other players (e.g. outsourcing, alliances, and joint ventures – i.e. collaborations) (Maine and Garnsey, 2006). Collaborations with other organizations can help new ventures share risks and therefore reduce the complexity (Chesbrough, 2003a, b). Hence, Marx *et al.* (2014) and Marx and Hsu (2015) identified that new ventures who aim to commercialize their technologies, but lack resources and capabilities, can pursue their ideal strategy by adopting interim co-operative strategies to access the needed complementarities. This consists in developing a temporary, not ideal, sub-strategy, such as launching the product briefly on the market to test the technology and get proof of it. Once the firm has proved the technology, it can aim to build partnerships with incumbents in the market. By doing so, the firm can achieve its ideal strategy. As such, the strategy can be exploited in the “Development” phase (Casadesus-Masanell and Ricart, 2010).

Independently from their experience, structural constraints, as well as cognitive biases, also contribute to the complexity in the technology commercialization, which can lead to high level of uncertainty (Gavetti *et al.*, 2012).

A BM that allows an organization to exploit its technological innovation (Chesbrough and Rosenbloom, 2002; Cortimiglia *et al.*, 2016) represents its implemented strategy (Casadesus-Masanell and Ricart, 2010). The BM can be exploited in the “Deployment” phase of technology commercialization.

Despite the wide range of definitions (e.g. Baden-Fuller and Haefliger, 2013; Chesbrough and Rosenbloom, 2002; Magretta, 2002; Osterwalder and Pigneur, 2010; Teece, 2010; Zott *et al.*, 2011), in general, BMs specify the key elements of the commercialization strategy, as they represent an organization’s essential activities in simplified form (Teece, 2010).

**Figure 1:**
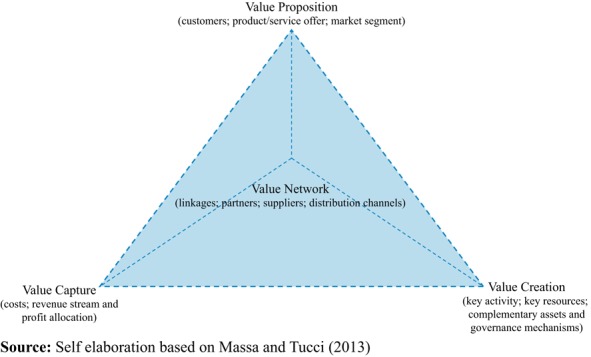
Network-centric business model

Overall, it is possible to distinguish two main approaches to the study of BMs: firm-centric (Magretta, 2002) and network-centric (Chesbrough and Rosenbloom, 2002). A firm-centric approach characterizes BMs along three dimensions: value creation, value proposition and value capture. The network-centric view of BMs includes the company network (see Figure 1).

This second perspective is consistent with an open firm’s strategy which has the advantage of increasing firm’s responsiveness to external influences (Vanhaverbeke and Roijakkers, 2013). It reflects how the firm collaborates with its ecosystem to develop and capture value (Massa and Tucci, 2013). In line with this view, Vanhaverbeke and Chesbrough (2014) see open BMs as “the situations where the innovating company relies on its partners’ competencies to jointly create value for customers and share that value according to agreements they have negotiated prior to the collaboration”.

### The commercialization of emerging technologies: the evolution of BMs

To navigate the complexity of the environment and successfully commercialize innovation, ventures need to be able to change and adapt their strategies and to reflect these in their BMs.

Thus, innovations do not happen only at the technology/service level, but also often occur at the BM level as a starting point for innovating a firm’s strategy (Mitchell and Coles, 2003; Zott *et al.*, 2011). Hence, the BM innovation can be seen as a comprehensive unit of analysis that allows managers/entrepreneurs to consider, at the same time, the most relevant endogenous and exogenous factors that would enable their organization to achieve competitive advantages and better performances (Datta *et al.*, 2015; Velu, 2015). Several scholars understand BMI as an instrument to understand a firm’s evolution, change and transformation (e.g. Chesbrough, 2010; Cortimiglia *et al.*, 2016; Demil and Lecocq, 2010; Dmitriev *et al.*, 2014; Hock, 2015; Johnson *et al.*, 2008). In fact, Schneider and Spieth (2013) argue that BMI has a positive influence on the firm’s ability to react to the continuous changes in the customers’ demands.

In general it is agreed that the BMI process is cyclical (e.g. Achtenhagen *et al.*, 2013; Chesbrough, 2010; Chesbrough and Rosenbloom, 2002; Demil and Lecocq, 2010; Dmitriev *et al.*, 2014; Lubik and Garnsey, 2016; Osterwalder and Pigneur, 2010; Simmons *et al.*, 2013; Teece, 2010) and involves two main phases: BM design (BMD) and BM reconfiguration (BMR) (Massa and Tucci, 2013). With similar meanings, Cortimiglia *et al.* (2016) referred to BMD and BM development, respectively. BMD is usually associated with the formation of new firms, and happens when an entrepreneur has to build, implement and validate a BM. BMR is the activity of improving BMs that already exist. The latter is typical of existing organizations.

Dmitriev *et al.* (2014) see BMI as a complex system of interactions that leads to a new BM, through a series of virtuous cycles, and via a “continuous process of conceptualizing value and organizing for value creation”. Sosna *et al.* (2010) showed that in a firm operating in a well-established industry, such as food, the development of new BMs occur through a trial-and-error process. This was confirmed by Lubik and Garnsey (2016), for science-based ventures. To date, it has been hypothesized that these BMI processes (design and reconfiguration) are instigated by certain “triggers” (discussed in the following section). What is known about the result of the BMI process (the “outcome”) is also summarized below.

Whilst, according to Johnson *et al.* (2008), a BMI cycle happens after new knowledge is gained, Demil and Lecocq (2010) and Dmitriev *et al.* (2014) showed that the BM cycles can be triggered by several factors, such as the interaction between and within the BM components, the interactions across the firm’s capabilities (e.g. market and technology) or inputs that come from the external environment. Generally, BMI triggers can be divided into three categories: external, internal and contextual (Demil and Lecocq, 2010). Internal triggers can be related to the effects of decisions that can affect the organizational system (e.g. decisions related to outsourcing a part of production). Changes in the BM can also be triggered by external factors such as changes to demand, new technological advancements or country-dependent environmental issues (Dmitriev *et al.*, 2014). Ultimately, the dynamism of a BM can be launched by contextual factors, such as the nature of an invention, the specific team of employees and the target market (Dmitriev *et al.*, 2014).

As a result of a BMI process, new BMs emerge. The resulting shape of BMs can be classified to identify archetypes (Massa and Tucci, 2013) – i.e. a general example of a BM. An example is “the Razor-and-blade BM”, which relies on “selling cheap razors to make customers buy its rather expensive blades” (Zott and Amit, 2010, p. 218). Taking an internal view on the BMI process, the difference between a pre-existing BM and a new one has also been highlighted by several authors (e.g. Brink and Holmén, 2009; Demil and Lecocq, 2010; Velu, 2015): the more radical the change in the BM component, the more the resulting BM is radical.

### A conceptual framework in the commercialization of technological innovation

**Figure 2:**
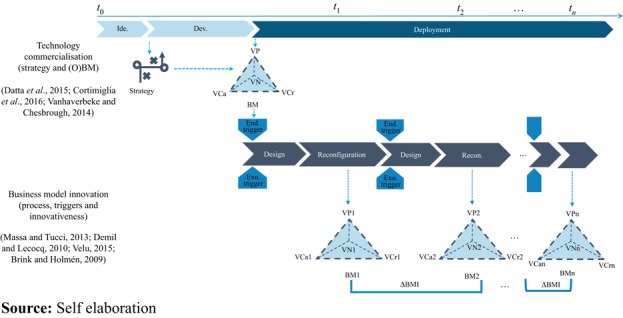
Conceptual framework: business model innovation in the technology commercialization process

A conceptual framework that synthesises the technology commercialization (strategy and (Open) BM and the BM innovation (process, triggers and degree of innovativeness) of an innovation is derived from the literature and reported in Figure 2.

In the case of emerging technologies, the commercialization process can be complex due to high technology and market uncertainty (Maine and Garnsey, 2006). The process becomes even more complex if it focuses on emerging technologies entering into established industries, due to the high barriers to entry and to the market saturation (Manzini *et al.*, 2016). Usually, emerging technologies are developed by new ventures (Maine *et al.*, 2012) that have to choose their commercialization strategies. For instance, new ventures developing the technology should choose their market. The most diffused options in the literature are niche (Davidow, 1986) or diversification (Nerkar and Shane, 2003) market strategies. This phase belongs to the “Development” stage of the technology commercialization process.

The exploitation of the strategies is realised in the formation of a BM within the “Deployment” commercialization phase (Datta *et al.*, 2015). To reduce the market and technology risks, and therefore the overall uncertainty, firms usually rely on collaborations, either to innovate (OI) and/or to commercialize (OBM). In this latter case, they assume a network-centric BM perspective with four BM dimensions (i.e. VProp, VCr, VCa and VNet).

As well as several other scholars, we see BMI as a cyclical process (see for instance Chesbrough and Rosenbloom, 2002; Teece, 2010; Demil and Lecocq, 2010; Osterwalder and Pigneur, 2010; Chesbrough, 2010; Simmons *et al.*, 2013; Dmitriev *et al.*, 2014; Lubik and Garnsey, 2016).

The BMI process belongs to the “Deployment” phase of the technology commercialization process (Datta *et al.*, 2015), where each cycle is triggered by exogenous or endogenous factors. While for exogenous triggers we mean all the factors that lead, for instance, to a new market opportunity and to technological advancements, for endogenous triggers we refer to all the factors related to a cognitive perspective (Hock, 2015).

Taking an internal BMI perspective (e.g. Brink and Holmén, 2009; Demil and Lecocq, 2010; Velu, 2015), within the framework one or more triggers initiate a BM cycle, and will influence one or more of the BM dimensions leading to a new BM (BM*n*), whose degree of innovation (ΔBM) will vary from incremental to radical (hence Incremental ⩽ΔBM ⩽ Radical). The more dimensions (VCa, VCr, VNet and VProp) change in a BM, the higher is the degree of novelty (radicalness) of the new BM (BM*n*+1). The process of BMI can be interpreted as an iterative process either of BMD and, sequentially, of BMR, or as a process of BMD and, separately, of BMR (Massa and Tucci, 2013). This process is continuous throughout the life of the firm, although there might be a time lapse between cycles (Dmitriev *et al.*, 2014). Once the BMI process ends, it results in a new BM that can assume various typologies and shapes (e.g. Aversa *et al.*, 2015; Casprini *et al.*, 2014; Cavalcante *et al.*, 2011).

Due to the complexity of the commercialization of emerging technologies, ventures firms can go through many cycles of development of their BM within the entire innovation process. As recently suggested by Lubik and Garnsey (2016), emerging-technologies-based ventures usually go through a “trial-and-error” process of learning to develop their BM. These companies can encounter many trigger-points that can start constant cycles of adjustments.

Research gap: whilst much has been highlighted about the possible strategic choices and the importance of BMI capabilities for ventures trying to enter an established market via the commercialization of a new technology (see Figure 2), examples of how companies respond to uncertainties and external triggers by adapting their BM are still scarce. This work aims to fill in this gap, by observing the evolution of BMs deployed by a venture which tries to penetrate the food industry with the commercialization of an emergent process technology (3D printing) and derive considerations which could deepen and add to the current theoretical understanding.

## Methodology

### Research design

The gap identified is an example of complex, current phenomena of which not much is known. In these cases, Yin (2009) suggests that a grounded approach, which employs a case study methodology, is the most suitable to investigate such phenomena. More specifically, a single case study method can support knowledge and theory building when it is used to extend the theory by determining if the theoretical set of circumstances (i.e. propositions) under study are correct, or if an alternative set of propositions would be more suitable instead (Siggelkow, 2007; Yin, 2009).

Single case studies are very useful in a variety of circumstances (Siggelkow, 2007) and have been particularly used in the BM literature, as this is an emergent theoretical area. For instance, Demil and Lecocq (2010) used a single case study to illustrate their RCOV framework used to link the static (i.e. BM) with the dynamic (i.e. BMI) approaches to look at BM development. Sosna *et al.* (2010) used a single case study to analyze the triggers and the antecedents that spur a new BMI cycle. Tongur and Engwall (2014) focused on the analysis of the inter-links between technology and BM. Toward this end, they narrowed their analysis to a single project. Velu and Stiles (2013) used an in-depth single case study to analyze how companies manage the decision-making processes in the circumstances in which emergent and existing BMs have to run in parallel. All the above highlighted studies have used a single case study, as this approach enabled the different authors to show a deeper level of detail and understanding of a certain phenomenon. This is an aim not pursuable with other methodological approaches.

We hence conducted in-depth, semi-structured interviews with one informant, asking him about past events (retrospective perspective), to test the suitability of the developed framework, and to identify advancement of this understanding. The interviews were supported by archival data analysis that included company documents, web news about the firm and the firm’s website.

### Sampling

We observed a UK-based new venture founded in 2009, IT IS 3D. IT IS 3D is a two-people technology-driven company focused on the distribution of Please, change to “3D Printing (3DP)” technologies in established industries. Its core business is in the education industry and only recently has IT IS 3D considered approaching the food industry. IT IS 3D represents a great example of an SME that has gone, and is going, through several BM cycles to access the food industry.

The CEO of IT IS 3D was interviewed twice in 2016. He is a serial entrepreneur who has spent most of his working life in engineering as the managing director of a business-to-business (B2B) distribution company specialized in commercializing emerging technology-based industrial applications, focusing lately on the education market.

The interviews were conducted face-to-face. Data were collected concerning the period starting from the founding of the firm to the present day (2016). Aware of the possible cognitive biases that this kind of approach could encounter, additional secondary data and notes were collected, as indicated above. The interviews were tape-recorded and later transcribed.

The first interview started with a brief account of the research project. Then, open-ended questions were used to gain more details, such as “tell me the story of your company? How has your business model been developing overtime?” (Minichiello *et al.*, 2008).

The second interview focused on clarifying the understanding gathered from the first interview and on validation of the BM cycle analysis.

### Data analysis

An inductive approach was adopted and a latent content analysis was performed (Mayan, 2009). This is described below.

IT IS 3D’s case has been analyzed through iterative phases. First, we coded all the data, primary and secondary. This process was iterated several times, until a clear account of the BM cycles emerged. Second, the data were categorized, according to BM building blocks and their constituent elements. Consistent with the open BM literature, the presence of a value network was held to indicate the openness of the model. As such, we indicated as a “closed model”, when the firm establishes a hierarchical relationship with suppliers and/or customers. A “partially open model” was defined as when the firm establishes a networked relationship, either on the supplier side or on the customer side. Otherwise, an “open model” was when the relationship with both the supplier as well as the customers is networked. Third, the data were tabulated in chronological order, following the development of each BM cycle. Fourth, broader themes such as the BM cycles, the BM changes and the BM strategy changes were derived (Mayan, 2009; Miles and Huberman, 1994). Fifth, after the analysis of the history of IT IS 3D, we looked for patterns and mechanisms in IT IS 3D BM dynamic processes. At the end of the analysis the observations derived from the case study were compared with the literature (Figure 2). The evidence from the observations of the case study was used to further develop insights captured in a new version of the theoretical framework (Figure 4).

## Findings: IT IS 3D history from a BM dynamics perspective

Currently IT IS 3D is a 3D printing distributor focusing on commercializing emerging technologies in niche segments of established industries. According to IT IS 3D’s CEO, this choice was determined by the commercialization potential of these kind of technologies “it is where the biggest opportunities lie. […] I want to do something that in 5 or 10 years will be very successful”.

### Background

IT IS 3D was born as a result of the failure of the firm where the founders were previously working. That company counted about 30 employees, but it unfortunately had to close, due to a misadventure with a fraudulent international partner. This firm operated according to a B2B distributor-based model for CNC and rapid prototyping equipment, as shown in Table I.

### IT IS 3D BMs innovation process: archetypes and timeline

IT IS 3D BM archetypes variation is shown in Table II.

#### BM1: distributor model

##### BM1*a*

IT IS 3D was founded in 2009 (at that time differently named), following the offer received by the founders to collaborate in the commercialization of a low-cost 3D printer. The new firm was configured as a low-cost 3D printer B2B distributor operating mainly in the education industry. The firm mainly replicated the BM adopted in the previous (failed) company, but wanted to substitute its previous transactional (closed) relationship with the manufacturer, with one where the new opportunities of business were explored more collaboratively (sharing the risks).

##### BM1b

About one year later (2010-2011), the 3D printer producer bypassed IT IS 3D and decided to directly commercialize its product. So IT IS 3D decided to internalize the production of 3D printers. They hired a 3D printing designer to produce their own low-cost 3D printer for schools. This is a change in the value creation building block, from external to internal. This change had a brief life, as, according to the CEO: “Having spent a lot of our money on developing a product which failed, we then had no money and no product. So, the only way to continue the business was to find another external supplier. And at that point we didn’t rethink our business, we just were looking for a quick solution to get us back into business without having to close the company”.

##### BM0

Giving up the production of their own 3D printing machine as “It took 12 months longer, than he (the 3D printer designer) and we have anticipated. And by the time it was launched, Chinese firms started coming into the market with machines that cost the same amount, but which are plug and play”, the firm reverted to the transactional-distributor model in the education industry (BM0) for a 3D printing equipment.

##### BM1c

“We limped along” said the CEO. So, at the end of 2014, IT IS 3D decided “trying to improve [their] existing business model [3D Printing technologies-based distributor], […]. Always, again, looking forward to new products […], thinking about new markets”. At this point they started, proactively, to consider distributing 3D printers in other industries. In 2014, IT IS 3D’s CEO came across a company that was producing a 3D printer that could be used to print food. This serendipitous finding spurred the following thought: “Maybe there is an opportunity in food, because it is still very early stage for 3D printing in food. And there are few competitors around the world”. Initially IT IS 3D tried to replicate the same BM (BM1a) with the low-cost 3D printer producer (e.g. close collaboration in commercializing the 3D food printer), but so far, the 3D food printer producer prefers to maintain a more transactional relationship.

#### BM2: consultancy (service) model

##### BM2a

At the same time IT IS 3D decided to revert to BM1a (2012-2013), they also decided to add a service BM on top of a distribution model. This new line of business was focused on delivering training and consulting in the education industry. The offering consisted in workshops to teach people how to use 3D printers and related equipment. “So we have done lots of school projects, spent the whole day with the school, and we would teach to the group of people how to use designing 3D, scanning 3D and seeing the results of their designs appearing printed”. This change indicates an “Outbound OI strategy” where the firm started capturing value from the expertise that they were giving away for free in the distribution model. “Instead of giving away a lot of intellectual property, which was knowledge about the market, knowledge about where the technology was going, knowledge about opportunities in the future. We started selling that”.

##### BM2b

When in 2014 IT IS 3D started considering new markets in which to sell 3D printers, the founders decided to expand the target market for the training and consulting activities in these industries too. In addition to the day workshop on how to use 3D printers, the firm started to offer lectures on the technology, the markets and the related potential business opportunities.

#### BM2-1: the linked BM

##### BM2b to BM1c

In the second half of 2015, IT IS 3D bought a prototype food printer with the intention to investigate new business opportunities in the food industry for both their consultancy (BM2b) and as a means to demonstrate the technology to customers interested in buying the 3D food printer (BM1c). Since, at that time the printer was not yet ready for distribution, the firm decided to use their consultancy BM2b to sell to potential customers, who were interested in using 3D printers in the food market, the chance to collaborate in testing the market opportunities for such technology. The value capture element of this BM would then be chosen after studying the potential customer business idea, depending on the interest of IT IS 3D: “if we thought the applications, the opportunities, would be big enough, we would do the test ourselves. If we didn’t, they would have to pay us to carry out the test and see whether it could be done”. Despite the high level of interest that IT IS 3D gained from the market, at the moment the work in this area is on hold, until the 3D printer will be finalized for distribution.

Recently in 2016, IT IS 3D requested consultancy from MBA students in order to understand how they can implement and expand their BM. IT IS 3D is “still trying to make a success of the same thing, but looking at different ways of making it happen”.

**Figure 3:**
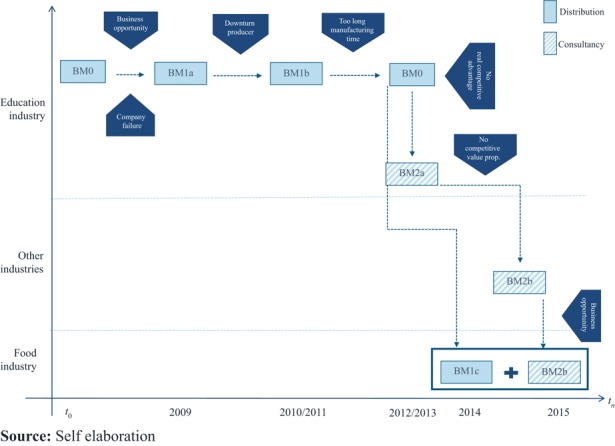
Sequence of business model and change triggers

Figure 3 visualizes IT IS 3D BM evolutions and the related triggers of change. On the *Y*-axis, it is possible to observe the market shifts, while on the *X*-axis there is the time line. Figure 3 also shows the twofold BMI strategies adopted by IT IS 3D: on the one hand, the BM evolution for the exploitation of 3DP within the education industry, on the other hand, the BM evolution following the exploration strategy to enter new markets.

### BMI process: influencing factors and logics

IT IS 3D is a relatively new venture, where the CEO leads the BMI process when he sees potential in new technologies/markets. The identification of new business opportunities often happened by chance (e.g. meeting people at workshops) rather than being deliberately sought in a structured way. “At the moment [the business development] is opportunity-based, rather than thinking ‘OK’, what are our core skills?”.

Despite BM additions and changes, IT IS 3D’s BM core structure has generally remained the same. For instance, when IT IS 3D was founded, it was in response to the forced closure of the previous business. The new venture started with the same BM archetype of the old company. The main difference was in the desire to change the network relationship with the technology producer for its commercialization.

According to the CEO, the main triggers that led to change are “Usually exogenous. Other things are happening over which we have no control”. For instance, a change happened when IT IS 3D switched from distributor to producer and vice versa. The first shift (BM1a-BM1b) was due to a decision by the printer producer to directly distribute its products. This led to changes in two out of the four BM building blocks (see Table II, i.e. VCr-VN). The second ΔBM (BM1b-BM0) was due to the lead-time to internally develop a product, increased competition and lack of liquidity. Also here, the ΔBM involved the VCr, which, however, remained closed (the relationship with the new equipment manufacturer was kept transactional, following the desire of the equipment manufacturer).

Another ΔBM happened when IT IS 3D looked to expand its market areas (BM0-BM1c), looking for new VPs, which could be supported through their products. This change originated from the lack of profits from one single market. VP change was replace with “also the main variation” in the design of the consultancy BM.

In the linked BM archetype, BM2b has been set to scope for new opportunities. IT IS 3D would run the test for scoping a new market opportunity, without asking for compensation, when the CEO expected potential future co-development of opportunities. So the firm was using an outbound OI strategy to explore new areas of business (i.e. VCr and VCa are open).

After six years of trial-and-error experimentations, the 3D printing-based distribution and consulting seems a formula for a stable source of income. “We would certainly continue with that core business”. However, the founders “are constantly on the lookout to how do we change our business model, in order to be successful?”.

## Discussion

To date, several studies have highlighted the importance of commercializing innovations through collaborations (e.g. Vanhaverbeke and Chesbrough, 2014; Massa and Tucci, 2013), by developing strategies (Grant, 2016; Casadesus-Masanell and Ricart, 2010) and BMs (e.g. Dmitriev *et al.*, 2014; Cortimiglia *et al.*, 2016) and in enhancing the ability of a new venture to face the uncertainties related to the commercialization of a new radical technology in a mature environment. However, previous studies did not show how these innovation processes take place (e.g. Probert *et al.*, 2013; Caiazza, 2015). This work aimed to contribute to this theoretical gap by providing empirical evidence on how BMs change when companies attempt to exploit an emerging technology in established industries.

To do so, we have looked at the history of a firm, IT IS 3D (from 2009 to 2016), concentrating on the BM development loops. Second, the analysis focused on the most relevant elements underlying the BMI process, compared with those derived from literature (see theoretical framework in Figure 2).

In the process of commercializing an emerging technology in an established industry, such as 3D printing in food, IT IS 3D decides the market strategies during the “Development” stage. In this phase, the firm under study tends to adopt differentiation strategies to explore new opportunities to get into the market, contrasting with what was stated by one stream of the innovation literature (Christensen, 1997; Christensen and Rosenbloom, 1995; Davidow, 1986). However, this finding was concurring to the market entry strategies for commercialization in mature industries (i.e. differentiation strategies), suggested by Grant (2016).

To reduce the market uncertainty emerging when a new entrant attempts to enter an existing market with a new radical technology it emerged that between the “Development” and the “Deployment” stage of the technology commercialization process, IT IS 3D develops interim strategies to identify the ideal one. Whilst this is partly in agreement with what highlighted by Marx *et al.* (2014) and Marx and Hsu (2015), according to which new ventures tend to develop interim strategies only to achieve the pre-defined final one, our observations contrast with these previous works. The reverse strategy process adopted by IT IS 3D has been introduced with an opposed arrow to the one designed in the original framework.

IT IS 3D’s CEO claims he is “not wedded to any one of the businesses, methodologies, the monetisation ideas that we have adopted in the past”. However, in line with what is argued by the cognitive school studying BMs (Martins, *et al.*, 2015); IT IS 3D mostly developed its BMs based on their previous experience as B2B equipment distributors. This observation shows that, potentially, the entrepreneur sees only some of the afforded BMs – i.e. the tactical manifestation of the strategy (Casadesus-Masanell and Ricart, 2010). However, this is not immediately obvious to the entrepreneur. When the CEO of IT IS 3D was asked why he was, more or less, following the same BM building blocks structure over time, he answered that “I am trying not to. [..] (Before) it never occurred me”. One explanation is the reduction of uncertainties. Faced with the complexity of both the market and the technology side (Maine *et al.*, 2012), it is conceivable that the firm would reduce the uncertainties by going back to BM archetypes that are familiar (e.g. this is what happened in the second BMI loop). Further changes were implemented to reduce uncertainties: IT IS 3D’s CEO highlighted several times that “In terms of being inside of the company, owning the technology and having a stake in that company, those are two of the key things that I have learned in my working life that I want to have”. This emphasizes the natural inclination to revert to the closed BMs.

**Figure 4:**
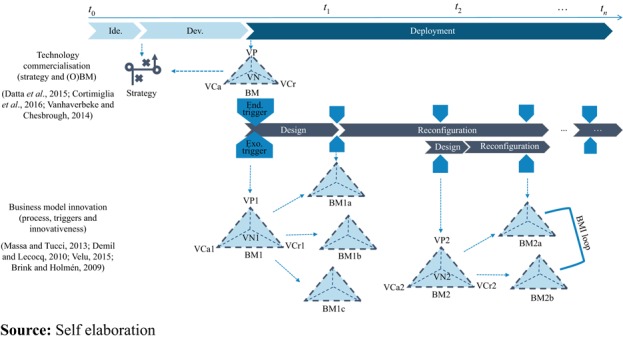
Implemented conceptual framework: business model innovation in the technology commercialization process

Furthermore, as only some elements of the BMs were changed at any one time (VCr and VN were successively revised, whilst the VCa model stayed mostly the same), it seems that not all the BM building blocks have the same relevance in the mind of an entrepreneur. If a new BM replicates the structure of an old BM, but for a new area of the market, then a loop in the BMI process emerged (see the example in Figure 4 from BM2a to BM2b). This finding has been introduced into the BM framework.

The case study also showed that BMR, which is typically attributed to established firms (Massa and Tucci, 2013), can also be found in start-ups. Furthermore, the case shows how the BMI process, which typically describes the BMI phases (BMD and BMR) in sequential order (Zott and Amit, 2010; Dmitriev *et al.*, 2014), is actually characterized by multiple repetitions and iterations of these two stages, and a firm develops its overall BM strategy running both types of BMI at the same time. Hence, both BMI processes (BMD and BMR) can co-exist for BM extension and revision (Cavalcante *et al.*, 2011).

Another finding emerged on the entity of the changes in BM (ΔBM). The literature-based framework highlighted that the radicalness of a BMI would depend on how many building blocks change. According to what has been observed in IT IS 3D’s case (see Table II and Figure 3), the radicalness regarded the whole strategy (which moved from an exploitative to a broader explorative one (March, 1991)) more than the BMs. The BM change, which this strategic shift initiated, was not, overall, a radical change. In other words, even important strategic changes (e.g. introducing a new line of business) do not reflect necessarily in a great ΔBM.

Although this is not the focus of this specific research, the proposed framework could also help in explaining how companies learn and acquire dynamic capabilities (Teece *et al.*, 1997) through repeated cycles of trial-and-error experimentation (Aldrich and Yang, 2014).

## Conclusions

To understand how BMs change when companies try to commercialize an emerging technology in established industries, this research followed three main steps. First, we built a conceptual framework from extant literature, which aimed to analyze the BM innovation cycles associated with the exploitation of emerging technologies. Second, we observed the data from the exploratory case study of IT IS 3D (a firm, which has started to exploit 3D printing in the food industry) though the lens for the initial conceptual framework. Finally, we integrated the findings that emerged from this case study with the theoretical framework designed from the literature. Overall, this work contributes to advancing the existing theoretical knowledge by showing how potential new entrants in an established market innovate, in the process of developing their BM for the commercialization of a new disruptive technology. The integrated framework also identified that:

1. More than one BM can exist at the same time. Hence, BMI processes (such as BMD and BMR) often co-exist and are run in parallel. So far the literature on the BMI process separates the BMD from the BMR. The first is typically associated with new ventures, while the second is associated with existing firms (Massa and Tucci, 2013). In contrast to the literature, this case shows that BMR can also happen for new ventures and that BMD and BMR can sometimes co-exist and run in parallel. These considerations are important for firms to tests the BMs whlst entering an established and conservative market such as the food industry.
2. To face the market uncertainty, a small firm could tend to adopt interim strategies and therefore BMs to identify the more suitable one to get into the market. The case of IT IS 3D show that, in order to reduce internal uncertainty, a firm could tend to adopt a closed and/or a familiar BM (e.g. establishing external, transactional-based relationships with manufacturers) by closing the value creation (i.e. how the firm create value) aspect of the BM. However, this case study also showed that explorative strategies could be pursued by opening (e.g. establishing more collaborative relationships with potential partners) the value creation/value proposition (i.e. the activity through which the firm aims to create and capture value) aspects of a BM, which would allow a firm to share risks. This combination of BM approaches could allow a firm to survive (if not flourish) whilst searching for a way to tackle a new market.
3. Even if significant changes in BM strategies are planned, these will not necessarily be reflected in the radicalness of the new BM.

By highlighting the above observations, this work can help managers to rationalize and review their BM development process for the commercialization of their new technology, by showing how the BM could be configured during the commercialization of an emerging technology in a mature industry. Similarly, it helps scholars appreciate that, although most literature often refers to a single BM and single strategies for technology commercialization, more can be combined and used to move forward in an uncertain transition. Specifically, from the case study of IS IT 3D it emerges that a firm could adopt interim BMs (i.e. open BMs to explore new opportunities and closed BMs in condition of internal uncertainty) to identify a more suitable one.

Further more, our observations could explain the incongruence in the suggestions from the strategic literature reported above and suggest that scholars might need to move from suggesting the “successful” strategies and BM configuration, instead taking into account a more dynamic and contingent view which describes the process of sustainably determining the best BM configuration to reduce the uncertainties in the penetration of established markets. Therefore, these observations can be extended to new ventures aiming to enter the food industry.

This work is exploratory and has several limitations. First, this is a single case study which has explored one instance of the phenomenon. Another limitation is consistent with the retrospective perspective adopted in the study. Even if the authors have mitigated this bias by collecting and integrating the data from the interviews with secondary data, it still poses a limitation, linked to the subjectivity of the informant in recollecting the IT IS 3D BM innovation sequence. Furthermore, only the opinion of the firm’s CEO was recorded. Hence, the results obtained here should provide the foundation for further studies testing of the framework on a broader sample of cases.

## Figures and Tables

**Table I:** Previous firm business model archetype

VP	VCr	VCa	VN
*BM0*			
Distributor of CNC machining and rapid prototyping equipment for industrial and educational needs	External manufacturers + internal knowledge	Margin on sold pieces of equipment. (The equipment were bought in advance and then re-sold at a higher price)	Closed model – hierarchical relationship with both suppliers and customers

**Table II:** Business model innovation dynamics

Distribution					Consultancy		Linked Consultancy to open new markets	
	BM1a	BM1b	BM0	BM1c	BM2a	BM2b	BM2b	BM1c
VP	Distributor of 3DP equipment for educational needs	Distributor of 3DP equipment for educational needs	Distributor of 3DP equipment for educational needs	Distributor of 3DP equipment for educational and *other markets (e.g. food) needs*	*Knowledge of 3DP technologies in education*	*Technical/Market* knowledge of 3DP in education – *industry*	Technical/Market knowledge of 3DP in education – industry – *food*	Distributor of 3DP equipment for educational as well as other markets (e.g. food) needs
VCr	External partner (3DP manufacturer) + internal knowledge	*Internal production*	*External manufacturers + internal knowledge*	External manufacturers + internal knowledge	*Internal knowledge*	Internal knowledge	*Internal knowledge – or – Networked with client*	External manufacturer
VCa	Margin on sold equipment (Buyers pay in advance)	Margin on sold equipment (Buyers pay in advance)	Margin on sold equipment (Buyers pay in advance)	Margin on sold equipment (Buyers pay in advance)	*Pay per time*	Pay per time	*Pay per test and advice* – otherwise – *Open (e.g. a percentage on every sold product/service co-created)*	Margin on equipment sold (Buyers pay in advance)
VN	Partially open model – *Open only for VCr (collaboration with provider of equipment to identify new opportunities)*	Closed model – *Hierarchical relationship with material suppliers and customers*	Closed model – Hierarchical relationship with equipment manufacturer, material suppliers and customers	Closed model – Hierarchical relationship with equipment manufacturer, material suppliers and customers	Closed model – Hierarchical relationship both with equipment manufacturers, material suppliers and customers	Closed model *–* Hierarchical relationship both with equipment manufacturers, material suppliers and customers	Closed model – Hierarchical relationship both with equipment manufacturer, material suppliers and customers – otherwise – Partially open model – Open only for VCr (external client in building business opportunities)	Closed model – Hierarchical relationship with equipment manufacturer, material suppliers and customers

**Note:** Changed BM building blocks from previous version are highlighted in italic
